# Post hospital admission blood lactate measurements are associated with mortality but not neurologic morbidity in children with cerebral malaria

**DOI:** 10.1186/s12936-024-04843-z

**Published:** 2024-01-19

**Authors:** Ronke Olowojesiku, Meredith G. Sherman, Amina M. Mukadam, Rami Imam, Kennedy M. Chastang, Karl B. Seydel, Alice M. Liomba, John R. Barber, Nicole F. O’Brien, Douglas G. Postels

**Affiliations:** 1 Department of Pediatrics, Children’s National Hospital, Washington, DC USA; 2 Global Health Initiative, Children’s National Hospital, Washington, DC USA; 3 University of Washington, Seattle, WA USA; 4 The George Washington University School of Medicine, Washington, DC USA; 5 Howard University, Washington, DC USA; 6 Michigan State University, East Lansing, MI USA; 7 Kamuzu University of Health Sciences, Blantyre Malaria Project, Blantyre, Malawi; 8 Division of Biostatistics and Study Methodology, Children’s National Research Institute, Washington, DC USA; 9 Division of Critical Care Medicine, Nationwide Children’s Hospital, Columbus, OH USA; 10 Division of Neurology, George Washington University/Children’s National Hospital, Washington, DC USA

**Keywords:** Malaria, Lactate, Paediatric, Africa, Malawi

## Abstract

**Background:**

In children with cerebral malaria (CM) admission blood lactate has previously guided intravenous fluid therapy and been validated as a prognostic biomarker associated with death. The usefulness of post-admission measurements of blood lactate in children with CM is less clear. The strength of association between blood lactate and neurological sequelae in CM survivors, as well as the optimal duration of post-admission measurements of blood lactate to identify children at higher risk of adverse outcomes is unknown.

**Methods:**

A retrospective cohort study of 1674 Malawian children with CM hospitalized from 2000 to 2018 who had blood lactate measurements every 6 h for the first 24 h after admission was performed. The strength of association between admission lactate or values measured at any time point in the first 24 h post-admission and outcomes (mortality and neurological morbidity in survivors) was estimated. The duration of time after admission that lactate remained a valid prognostic biomarker was assessed.

**Results:**

When lactate is analysed as a continuous variable, children with CM who have higher values at admission have a 1.05-fold higher odds (95% CI 0.99–1.11) of death compared to those with lower lactate values. Children with higher blood lactate at 6 h have 1.16-fold higher odds (95% CI 1.09–1.23) of death, compared to those with lower values. If lactate levels are dichotomized into hyperlactataemic (lactate > 5.0 mmol/L) or not, the strength of association between admission lactate and mortality increases (OR = 2.49, 95% CI 1.47–4.22). Blood lactate levels obtained after 18 h post-admission are not associated with outcomes. Similarly, the change in lactate concentrations through time during the first 24 h of hospital admission is not associated with outcomes. Blood lactate during hospitalization is not associated with adverse neurologic outcomes in CM survivors.

**Conclusions:**

In children with CM, blood lactate is associated with death but not neurologic morbidity in survivors. To comprehensively estimate prognosis, blood lactate in children with CM should be assessed at admission and for 18 h afterwards.

## Background

Despite decades of progress in decreasing disease incidence, malaria continues to have a significant public health impact, with an estimated 241 million cases and 627,000 deaths annually [1]. Children under 5 years old account for two-thirds of these deaths [1]. Clinical disease is classified as either uncomplicated or severe [2]. Cerebral malaria (CM) is the severe malaria syndrome with the highest mortality risk [3]. CM is clinically defined as an otherwise unexplained coma in someone with asexual *Plasmodium* parasitaemia [3]. Universally fatal if untreated, with effective anti-malarial treatment and close supportive care the mortality risk in CM is 15–20% [3, 4]. Among CM survivors, 10 to 30% have cognitive, neurologic, and/or behavioural sequelae [5, 6].

Lactate is the primary measurable result of anaerobic metabolism. In severe illness, when blood oxygen levels fail to meet tissue oxygen demands, anaerobic metabolism begins, producing lactate. In CM and other severe malaria syndromes, the origin of elevated blood lactate is likely multifactorial. Proposed mechanisms include parasite production, decreased host perfusion secondary to parasite sequestration (and secondary anaerobic metabolism), and anaemia resulting in lower oxygen carrying capacity [7, 8]. In both malarial and non-malarial severe illness, addressing this oxygenation deficit through resuscitation has been a therapeutic goal.

Published clinical care guidelines for children with CM recommend routine measurement of blood lactate at admission to aid in patient care [9, 10]. The World Health Organization (WHO) additionally recommends measurement of blood lactate 4 h after presentation to care and/or four hours after fluid resuscitation [11]. As hyperlactataemia is known to be associated with poor outcomes [12–16], prior standard clinical practice often included bolus administration of intravenous fluids to children with CM with elevated blood lactate who may have had other signs of impaired perfusion. However, the publication of the results of the Fluid Expansion as Supportive Therapy (FEAST) clinical trial in 2011 revealed increased mortality in African children with impaired perfusion who received bolus intravenous fluids [17]. Consequently, administering bolus intravenous fluids to African children with febrile coma (including CM) with signs or symptoms of impaired perfusion and hypovolaemia, including hyperlactataemia, has largely ceased. Use of other therapies to address hyperlactataemia have not been successful. Clinical trials of dichloroacetate, used to target hyperlactataemia, were not successful in modifying outcomes [18–22].

While the use of blood lactate to target fluid resuscitation in African children with CM has waned, it remains an important prognostic biomarker. In severe malaria, an admission blood lactate greater than 5.0 mmol/L is a risk factor for mortality [23]. In Bangladeshi adults with severe malaria, measures of blood lactate at 8- and 12-h post-admission were identified as good surrogate endpoints for studies on anti-malarials [24]. What remains unclear is whether lactate measurement in children with CM or other severe malaria syndromes is a valid prognostic biomarker post-admission and, if so, how long after admission blood lactate continues to be associated with outcomes.

In the clinical care of children with CM, if blood lactate is measured, it is commonly done so only at admission. Though associations between admission laboratory factors and outcomes are important, it is possible that trends in prognostic biomarkers through time may be more informative than a single measurement collected at the time of hospital presentation. In children with non-malarial illnesses (e.g. pneumonia and sepsis) and hyperlactataemia, a higher rate of post-admission lactate clearance is associated with decreased mortality risk [24–29]. Whether elevated post-admission lactate or trends in this laboratory value through time are also associated with outcomes in paediatric CM is unknown. To evaluate the optimal duration of measurements of blood lactate in children with CM, the strength of association of outcomes (mortality or neurological morbidity in survivors) with lactate trends through time for the first 24 h after hospitalization was assessed.

## Methods

A retrospective cohort study of children aged 6 months to 14 years old admitted to Queen Elizabeth Central Hospital (QECH) in Blantyre, Malawi with clinical CM, defined as a Blantyre Coma Scale (BCS) ≤ 2, asexual forms of *Plasmodium falciparum* on peripheral blood smear, and no other known aetiology of coma [3] was performed. The cohort data came from children with clinical CM admitted to a long-standing observational study of CM pathogenesis between September 2000 and June 2018. Guardians of all study participants consented to enrollment in the parent observational study, which was approved by the Institutional Review Boards of Michigan State University (USA) and the University of Malawi College of Medicine (Malawi). At the time of enrollment, guardians consented to the future use of deidentified data in secondary analyses, such as those used here. Ethical approval for this secondary analysis was provided by Children’s National Research Institute (Washington, DC, USA).

Participants were hospitalized on the QECH Paediatric Research Ward, an inpatient unit specialized in the evaluation and clinical care of children with febrile coma, including CM. On admission, children were clinically stabilized, begun on intravenous fluids at maintenance rates, and administered intravenous anti-malarials, quinine through 2013, and artesunate thereafter. Children treated with quinine received a loading dose of 20 mg/kg infused over 4 h followed by three doses of 10 mg/kg every 12 h. In 2014 and thereafter, anti-malarial treatment was initiated with artesunate given as three doses per Malawi Ministry of Health dosing guidelines. After 24 h of intravenous anti-malarials, therapy was transitioned to a 3-day course of enteral lumefantrine–artemether, either orally or via nasogastric tube for patients who remained unconscious. Those who received lumefantrine–artemether by nasogastric tube were transitioned to oral medications when clinically appropriate.

Supportive care included anticonvulsants for clinical or non-convulsive seizures (as determined by admission 30-minute electroencephalogram), blood transfusions at 10 mL/kg of packed red blood cells for those with a haematocrit less than 15%, and enteric feeds via nasogastric tube, typically begun 24 h after admission. Mechanical ventilation was not available during the study period.

Before publication of the FEAST clinical trial in 2011 [17] most patients with signs of hypovolaemia (e.g. decreased skin turgor, capillary refill more than 3 s, tachycardia, and/or gallop rhythm on cardiac auscultation) were administered normal saline 10–20 mL/kg intravenously over 1 h. After 2011, bolus fluid administration for those with signs of hypovolaemia was no longer standard practice.

Patient demographics, history, and physical examination findings were collected on admission. Baseline laboratory studies included a thick and thin smear for malaria parasites, full blood count (used for the calculation of circulating parasitaemia), blood culture, lumbar puncture, and point of care testing for glucose and lactate using Accuchek (Roche, Indianapolis, Indiana, USA) and LactatePro (Arkray, Mumbai, India), respectively. After admission, blood was obtained every 6 h until two consecutive thick and thin smears were negative for malaria parasites. Point of care testing for lactate and glucose were obtained every 6 h for the first 24 to 48 h after admission.

At the time of hospital discharge, survivors were assessed for the presence of neurologic sequelae by trained clinicians. Children with neurologic sequelae were classified as Survived with Neurologic Sequelae based on a standardized neurologic examination and/or family report of new abnormalities in hearing, vision, cognition, movement, tone, or development.

### Statistical analysis

Hyperlactataemia was defined as a blood lactate greater than 5 mmol/L [16, 17, 30]. Summary statistics were calculated for patient demographics and baseline laboratory values as means and standard deviations for continuous variables, and counts and frequencies for categorical variables. Characteristics were compared between children who died to those who survived. In a separate analysis, these characteristics were compared in those who survived and returned to their baseline to those who survived with neurologic sequelae. Comparisons were made using the Wilcoxon rank test for continuous characteristics, or χ^2^ tests for categorical variables. Peripheral parasite densities were logarithmically transformed before comparison. The anti-malarial used (quinine or artesunate) was controlled for in all analyses.

Significant terms with a *p* < 0.10 from univariate comparisons were then used in their respective multivariate logistic regression models with death or neurologic sequelae in survivors as the outcomes of interest. In these models, lactate values in longitudinal (post-admission) time points were included in addition to the admission status. Due to missing data after 24 h post-admission, only lactate measurements in the first 24 h were included in multivariate models. In multivariate models, analyses were confined to children who had lactate measurements recorded at admission and at least one time point afterwards. One multivariate model was created using lactate as a continuous variable and a second multivariate model was created using dichotomized lactate values into hyperlactataemia (blood lactate > 5.0 mmol/L) or no hyperlactataemia. In models where blood lactate values were dichotomized, a covariate for “ever hyperlactataemic” was created and was defined as a child with blood lactate at any time point in the 6–24 h post-admission greater than 5 mmol/L. Additionally, the slope of the lactate clearance line as well as a dichotomized trend of serum lactate in the first 24 h after admission (lactate at 24 h greater than, equal to, or less than the admission value) were evaluated. In multivariate models, odds ratios and 95% CI for the addition of these longitudinal lactate measurements were estimated.

Mortality and morbidity risk in children who were newly hyperlactatemic at any time point in the first 24 h of admission were assessed. To assess whether the administration of a fluid bolus is associated with a decrease in serum lactate, a two-sample t-test/Wilcoxon Rank Sum test was performed. The effect of fluid bolus on survival status was analysed by a chi-squared test to assess whether bolus given was independent of the outcome and then added to a multivariate logistic regression model. Analyses were performed using SAS version 9.4 (Cary, North Carolina, USA) with 2-sided tests.

## Results

A total of 1674 children with CM and at least one lactate measurement were admitted to the Paediatric Research Ward between September 2000 and June 2018. Of these, 272 (16.2%) died. Among the 1402 survivors, 152 (10.8%) had neurologic sequelae.

In univariate analysis, multiple admission demographic, clinical, and laboratory factors were associated with adverse outcomes (Table 1). Children with a lower BCS, higher peripheral parasite count, higher admission white blood cell count, and higher blood lactate all had an increased likelihood of death, compared to children who did not have these abnormalities. In survivors, children who were younger, had a lower admission BCS, longer coma duration, and higher admission white blood cell count all had higher rates of adverse neurologic outcomes, compared to those without these characteristics. Children who received a crystalloid fluid bolus or multiple blood transfusions were more likely to die compared to those who did not.

**Table 1:** Admission demographic, clinical information, and outcomes of children with cerebral malaria

Demographic or laboratory measure	Survived without neurologic sequelae (*N* = 1250)	Survived with neurologic sequalae (*N* = 152)	Died (*N* = 272)	P value for difference (died vs. survived)	P value for difference (in survivors, normal vs. neurologic sequelae)
Age, months, mean (SD)	47.2 (29.8)	42.0 (26.5)	44.7 (27.3)	0.463	0.037
Sex, N (%)					
Male	605 (48%)	75 (49.3%)	120 (44.4%)	0.218	0.833
Female	644 (52%)	77 (50.7%)	150 (55.6%)		
Blantyre Coma Scale at admission, N (%)					
0	124 (9.9%)	25 (16.5%)	69 (25.3%)	< 0.001	< 0.001
1	480 (38.4%)	77 (50.7%)	122 (44.9%)		
2	646 (51.7%)	50 (32.9%)	81 (29.8%)		
Coma duration (hours), mean (SD)	12.3 (15.8)	13.9 (16.1)	11.0 (11.9)	0.990	0.008
Parasite count, mean (SD), log (10)	4.5 (1.2)	4.5 (1.1)	4.7 (1.1)	0.003	0.925
Haematocrit %, mean (SD)	22.4 (7.2)	23.7 (8.6)	22.6 (8.4)	0.858	0.165
Severe malarial anaemia (Hct < 15%), N (%)	180 (14.5%)	20 (13.5%)	50 (18.5%)	0.087	0.737
White blood cell count, mean (SD)	11.0 (8.0)	12.5 (6.7)	15.0 (11.3)	< 0.001	< 0.001
Lactate (mmol/L), mean (SD)	6.3 (4.4)	6.6 (4.6)	9.2 (4.9)	< 0.001	0.534
Hyperlactataemia, N (%)	496 (49%)	61 (52%)	162 (78%)	< 0.001	0.555
Glucose (mmol/L), mean (SD)	6.6 (3.4)	6.7 (4.3)	6.7 (4.9)	0.179	0.483
Received blood transfusion, N (%)	445 (36%)	55 (36%)	92 (34%)	0.561	0.887
Received > 1 blood transfusion	25 (2%)	5 (3%)	18 (7%)	< 0.001	0.300
Received crystalloid bolus, N (%)	153 (12%)	25 (16%)	81 (30%)	< 0.001	0.141
Received > 1 bolus	25 (2.0%)	5 (3.3%)	18 (6.6%)	< 0.001	0.300

In multivariate modeling using death as the outcome of interest and blood lactate as a continuous variable, similar clinical and demographic variables were independently associated with death. For each 1 mmol/L increase in blood lactate, the odds of death increased by 1.05 (95% confidence interval [CI]: 0.99–1.11). At 6 h after admission, the strength of association between blood lactate and death was higher with 1.16-fold higher odds of death (95% CI: 1.09–1.23) for each 1 mmol/L blood lactate increase (Table 2). Addition of other post-admission lactate values (slope of lactate values through 24 h, dichotomized comparisons of admission and 24-h lactate, lactate values after 6 h) were not retained in multivariate model selection. Multivariate modelling with adverse neurologic sequelae in survivors as the outcome of interest was not performed because lactate was not associated with this outcome on univariate analysis.

**Table 2:** Independent association of demographic, clinical, and laboratory values with death in children with CM using blood lactate as a continuous independent variable

	Univariate		Backwards selection (P-stay < 0.1)	
	OR	95% CI	OR	95% CI
Age, months, mean (SD)	0.99	(0.99–1.00)		
Sex, N (%)				
Male	1	(Ref)	1	(Ref)
Female	1.37	(0.94–2.01)	1.72	(1.12–2.66)
Blantyre Coma Scale at admission, N (%)				
0	1	(Ref)	1	(Ref)
1	0.53	(0.31–0.91)	0.71	(0.39–1.32)
2	0.30	(0.17–0.53)	0.45	(0.24–0.84)
Parasite count, mean (SD), log (10)	1.28	(1.07–1.53)		
Admission haematocrit %, mean (SD)	1.03	(1.00–1.05)	1.06	(1.03–1.05)
Admission white blood cell count, mean (SD)	1.04	(1.02–1.06)	1.02	(1.00-1.05)
Admission Lactate (mmol/L), mean (SD)	1.12	(1.07–1.16)	1.05	(0.99–1.11)
6 h Lactate (mmol/L), mean (SD)	1.20	(1.14–1.26)	1.16	(1.09–1.23)
Glucose (mmol/L), mean (SD)	1.01	(0.96–1.07)		
Received blood transfusion, N (%)	1.18	(0.81–1.72)		
Received > 1 blood transfusion	3.38	(1.62–7.05)		
Received crystalloid bolus, N (%)	3.29	(2.22–4.87)	1.80	(1.07–3.03)
Received > 1 bolus	3.38	(1.62–7.05)		

When blood lactate values were dichotomized into hyperlactataemia or no hyperlactataemia (using 5 mmol/L as a cutoff), the results of multivariate modeling changed (Table 3). Those who had hyperlactataemia on admission had a 2.49-fold higher odds of death (95% CI 1.47–4.22) compared to those who did not. If hyperlactataemia was present at 6 h after admission, the odds of death were 2.20-fold higher (95% CI 0.91–5.32) compared to those without hyperlactataemia at this time point. Children who receive crystalloid boluses had a higher risk of death compared to those who did not (Table 3). Children administered blood had no change in mortality risk.

**Table 3:** Independent association of demographic, clinical, and laboratory values with death in children with CM when blood lactate values are dichotomized

	Univariate		Backwards selection (P-stay < 0.1)	
	OR	95% CI	OR	95% CI
Age, months, mean (SD)	0.99	(0.99–1.00)		
Sex, N (%)				
Male	1	(Ref)	1	(Ref)
Female	1.37	(0.94–2.01)	1.63	(1.07–2.48)
Blantyre Coma Scale at admission, N (%)				
0	1	(Ref)	1	(Ref)
1	0.53	(0.31–0.91)	0.67	(0.37–1.21)
2	0.30	(0.17–0.53)	0.42	(0.23–0.77)
Parasite count, mean (SD), log (10)	1.28	(1.07–1.53)		
Admission haematocrit %, mean (SD)	1.03	(1.00-1.05)	1.04	(1.01–1.07)
Admission white blood cell count, mean (SD)	1.04	(1.02–1.06)	1.03	(1.01–1.05)
Admission hyperlactataemia	2.95	(1.94–4.50)	2.49	(1.47–4.22)
6-Hour hyperlactataemia	0.99	(0.46–2.12)	2.20	(0.91–5.32)
Glucose (mmol/L), mean (SD)	1.01	(0.96–1.07)		
Received blood transfusion, N (%)	1.18	(0.81–1.72)		
Received > 1 blood transfusion	3.38	(1.62–7.05)		
Received crystalloid bolus, N (%)	3.29	(2.22–4.87)	2.40	(1.52–3.79)
Received > 1 bolus	3.38	(1.62–7.05)		

**Fig. 1:**
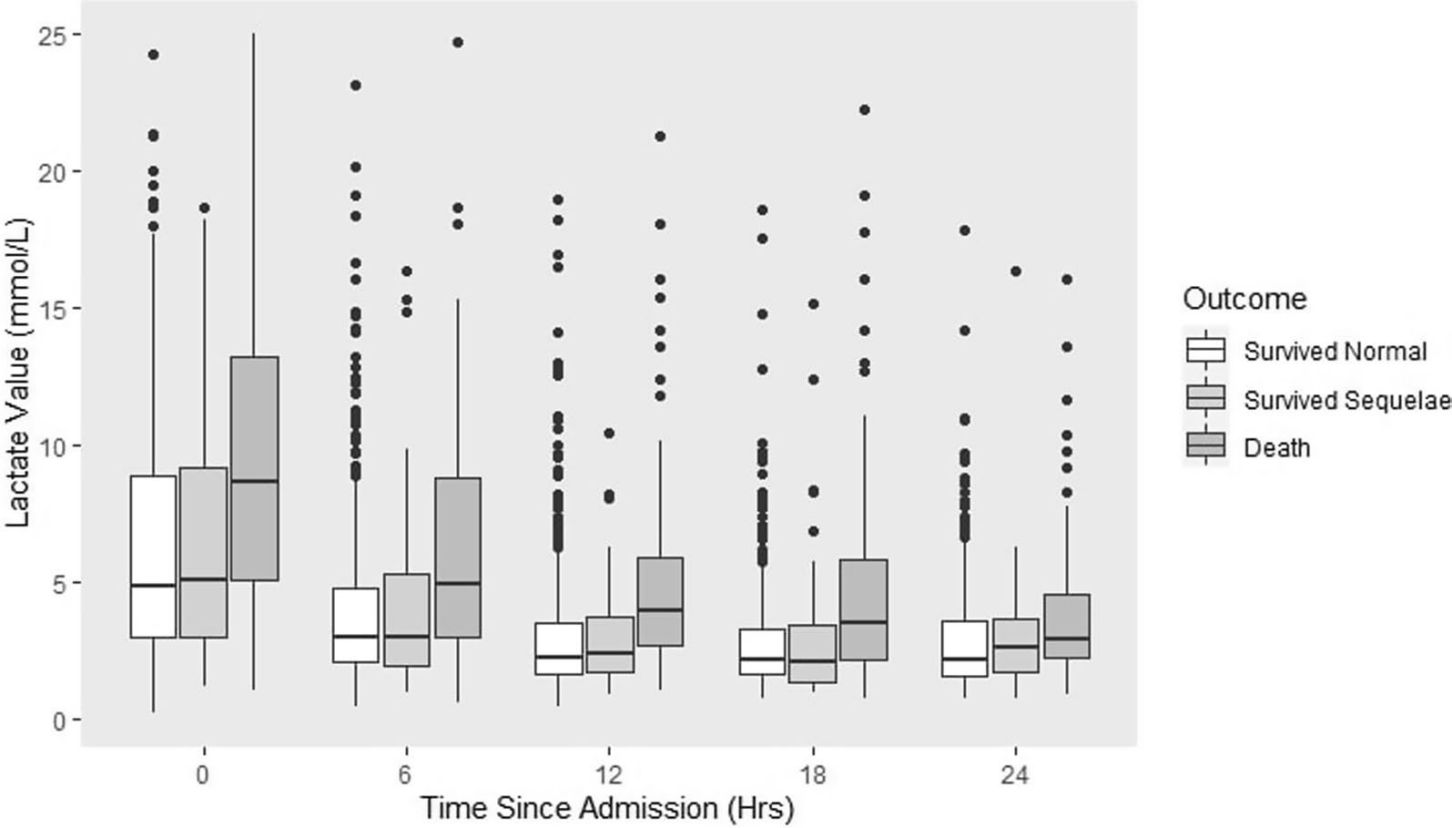
Box and whisker plot of blood lactate values in the first 24 h of hospital admission in children with cerebral malaria

Mean blood lactate values decreased through time across all groups, though remained highest among those who died (Fig. 1). Results of longitudinal measurements of blood lactate revealed that new episodes of hyperlactataemia were detected throughout the first 24 h after hospital admission (Table 4). Mortality and morbidity rates in those with hyperlactataemia detected at admission and at 6, 12, and 18 h post-admission were higher than those without hyperlactataemia. At 24 h post-admission, those with newly detected hyperlactataemia had a similar risk of adverse outcomes compared to those with normal lactate.

**Table 4:** Incidence of hyperlactataemia in the first 24 h of hospital admission among children with cerebral malaria

Time (hours)	N at beginning of time interval^a^	Deaths before next time point	Hyperlactataemia (n)	Number of those with hyperlactataemia without previous episodes of hyperlactataemia	Mortality rate of those with hyperlactataemia (%)	Mortality rate of those without hyperlactataemia (%)	p-value	Morbidity rate of those with hyperlactataemia (%)
0 (admit)	1336	61	717	NA	22	8	< 0.001	49
6	1043	28	286	67	22	9	< 0.001	25
12	977	17	142	21	22	8	< 0.001	13
18	929	23	119	24	22	6	< 0.01	10
24	864	25	112	30	12^b^	6	0.022	10

^a^Participants at the beginning of time interval are those who were alive and had lactate measurements recorded at that time point

^b^At 24 h post-admission, the mortality rate of those with elevated blood lactate falls below the mortality fate of the overall cerebral malaria cohort

## Discussion

In children with CM, elevated blood lactate is associated with increased odds of death at admission and 6, 12, and 18 h post-admission. Increased blood lactate is not associated with an increased odds of adverse neurologic outcomes in CM survivors. In considering the reason as to why elevated blood lactate at later time points post-admission is associated with increased mortality, the persistence of hyperlactataemia after presentation to the hospital and initial resuscitation efforts likely speaks to the failure of initial interventions. Congruent with this study’s results, in Indian children with sepsis, blood lactate levels at 6 h after admission are more strongly associated with mortality than levels at admission [29].

In both univariate and multivariate analyses, admission lactate and values of lactate during the first 24 h after admission are not associated with adverse neurologic outcomes in CM survivors. This study’s findings are similar to those in previously published studies evaluating associations between exposures and neurologic sequelae in CM survivors [4–6, 10]. The association of elevated lactate with mortality but not with neurologic morbidity supports the hypothesis that the pathophysiological pathway between infection and death may be different than that between infection and neurologic morbidity. The lack of congruence between independent risk factors for mortality and neurological morbidity in survivors, including lactate, supports the hypothesis that in children with CM, these two outcomes are not a continuum.

This study has many strengths, foremost its large sample size. This is the largest study of blood lactate in paediatric CM to date. Additionally, the personnel caring for the children over the 18-year data collection period did not substantially vary through time, decreasing the likelihood of temporal bias. However, the use of bolus crystalloid administration for children with clinical signs of hypovolaemia or elevated lactate changed after publication of the FEAST clinical trial. Though no difference in recommendations for the duration of lactate measurements in children enrolled before and after FEAST’s publication were found, it is possible that the smaller number of children in each group (pre-FEAST and post-FEAST) limited study power to detect associations between exposures and outcomes.

This study has several limitations. Clinical laboratory confirmation of point of care testing of lactate values was unavailable at the hospital where this study was conducted. As the blood lactate levels reported were from finger stick samples as opposed to free-flowing venous samples, there may have been false elevations in blood lactate, especially in children who were hypovolaemic. Additionally, blood lactate measurements, if any, that were taken prior to arrival on the Paediatric Research Ward were not included in these analyses. It is possible that children may have received intravenous fluid boluses before arrival at the study site, a referral hospital. As the administration of bolus fluids prior to hospital admission could not be confirmed, these analyses may not truly reflect the amount of intravenous fluid resuscitation received by study participants. Future prospective cohort studies evaluating high blood lactate as a prognostic biomarker in paediatric CM should consider enrolling children from the time they present for healthcare.

Demographic, clinical, and laboratory factors associated with neurologic sequelae were evaluated at the time of hospital discharge. Since neurologic sequelae may either resolve (e.g., motor disorders) or newly appear (e.g., developmental delay, epilepsy) post-hospitalization, it is likely more meaningful to evaluate associations between exposures and long-term neurologic abnormalities. As post-discharge neurologic and developmental assessments were available for only a small proportion of the children in the cohort, they were not included in these analyses. The results of this study should be confirmed with prospective data collection.

## Conclusions

In paediatric CM, high blood lactate at the time of admission or 6 h after admission is independently associated with death but not with neurologic sequelae in CM survivors. When analysed as a dichotomous variable, blood lactate is associated with death at the time of admission and up to 18 h afterwards. Neither trends in blood lactate through time nor blood lactate levels obtained after 18 h post-admission were associated with death. Neither blood lactate values in the first 24 h after hospitalization nor the trends in lactate through time are associated with adverse neurologic outcomes in CM survivors. To accurately assess prognosis in a child with CM, blood lactate should be measured at admission and 6, 12, and 18 h afterwards. Given blood lactate’s limited utility as a decision point for targeted intervention, measurements longer than 18 h post-admission are unlikely to be of value.

## Data Availability

The datasets used and/or analysed during the current study are available from the corresponding author upon reasonable request.

## Abbreviations

BCS: Blantyre Coma Scale
CI: Confidence interval
CM: Cerebral malaria
FEAST: Fluid Expansion as Supportive Therapy
QECH: Queen Elizabeth Central Hospital
